# Impact of regular magnetic resonance imaging follow-up after stereotactic radiotherapy to the surgical cavity in patients with one to three brain metastases

**DOI:** 10.1186/s13014-019-1252-x

**Published:** 2019-03-14

**Authors:** N. Bachmann, D. Leiser, E. Ermis, S. Vulcu, P. Schucht, A. Raabe, D. M. Aebersold, E. Herrmann

**Affiliations:** 1 Department of Radiation Oncology, Inselspital, Bern University Hospital and University of Bern, Freiburgstrasse 18, CH-3010 Bern, Switzerland; 2 Department of Neurosurgery, Inselspital, Bern University Hospital and University of Bern, Bern, Switzerland

**Keywords:** Brain metastases, Surgical cavity, Tumor bed, Magnetic resonance imaging, MRI, Radiosurgery, Stereotactic radiotherapy, Follow-up, Surveillance, Monitoring

## Abstract

**Background:**

Administering stereotactic radiotherapy to the surgical cavity and thus omitting postoperative whole brain radiotherapy (WBRT) is a favored strategy in limited metastatic brain disease. Little is known about the impact of regular magnetic resonance imaging follow-up (MRI FU) in such patient cohorts. The aim of this study is to examine the impact of regular MRI FU and to report the oncological outcomes of patients with one to three brain metastases (BMs) treated with stereotactic radiosurgery (SRS) or hypo-fractionated stereotactic radiotherapy (HFSRT) to the surgical cavity.

**Methods:**

We retrospectively analyzed patients who received SRS or HFSRT to the surgical cavity after resection of one to two BMs. Additional, non-resected BMs were managed with SRS alone. Survival was estimated by the Kaplan-Meier method. Prognostic factors were examined with the log-rank test and Cox proportional hazards model. Regular MRI FU was defined as performing a brain MRI 3 months after radiotherapy (RT) and/or performing ≥1 brain MRI per 180 days. Primary endpoint was local control (LC). Secondary endpoints were distant brain control (DBC), overall survival (OS) and the correlation between regular MRI FU and overall survival (OS), symptom-free survival (SFS), deferment of WBRT and WBRT-free survival (WFS).

**Results:**

Overall, 75 patients were enrolled. One, 2 and 3 BMs were seen in 63 (84%), 11 (15%) and 1 (1%) patients, respectively. Forty (53%) patients underwent MRI FU 3 months after RT and 38 (51%) patients received ≥1 brain MRI per 180 days. Median OS was 19.4 months (95% CI: 13.2–25.6 months). Actuarial LC, DBC and OS at 1 year were 72% (95% CI: 60–83%), 60% (95% CI: 48–72%) and 66% (95% CI: 53–76%), respectively. A planning target volume > 15 cm^3^ (*p* = 0.01), Graded Prognostic Assessment (GPA) score (*p* = 0.001) and residual tumor after surgery (*p* = 0.008) were prognostic for decreased OS in multivariate analysis. No significant correlation between MRI FU at 3 months and OS (*p* = 0.462), SFS (*p* = 0.536), WFS (*p* = 0.407) or deferment of WBRT (*p* = 0.955) was seen. Likewise, performing ≥1 MRI per 180 days had no significant impact on OS (*p* = 0.954), SFS (*p* = 0.196), WFS (*p* = 0.308) or deferment of WBRT (*p* = 0.268).

**Conclusion:**

Our results regarding oncological outcomes consist with the current data from the literature. Surprisingly, regular MRI FU did not result in increased OS, SFS, WFS or deferment of WBRT in our cohort consisting mainly of patients with a single and resected BM. Therefore, the impact of regular MRI FU needs prospective evaluation.

**Trial registration:**

Project ID: 2017–00033, retrospectively registered.

## Introduction

Brain metastases (BMs) are the most common tumors in the central nervous system (CNS) in adults [1]. Population-based studies show incidence rates of brain metastases ranging from 8.3 to 14.3 per 100,000 population. Among cancer patients, the incidence ranges from 8.5–9.6% [2], and 20 to 40% of patients with cancer develop brain metastases during the course of their disease [3]. Since the past two decades the incidence of BM is rising as a consequence of increased availability of magnetic resonance imaging (MRI) [4] and longer survival from primary cancers, due to new and more effective systemic therapy options [2]. The most common primary tumors to metastasize to the brain are lung cancer (50–60%), breast cancer (15–20%) and melanoma (5–10%), while tumors of the gastrointestinal tract and renal cell carcinomas (RCC) are less common [1, 5]. Most patients present with oligometastatic brain disease, which is defined as a limited number (usually 1 to 3) of intracranial metastases [6]. Surgery, radiosurgery, and whole-brain radiotherapy (WBRT) are the main treatment options depending on individual disease characteristics [7]. Recently, two randomized trials have been published [6, 8], addressing the fact that stereotactic radiosurgery (SRS) alone may be a preferred strategy for a limited number of unresected and resected brain metastases. No survival difference in the SRS alone vs. the WBRT group, but less cognitive deterioration as well as a higher quality of life (QoL) at 3 months with SRS alone was shown in both trials. On the other hand, time to intracranial failure was significantly shorter for SRS alone compared with SRS plus WBRT in both trials. The 6- and 12-month local and distant tumor control rates were also significantly higher in patients who received WBRT (*p* < 0.001). Additionally, fewer patients underwent salvage therapy after SRS plus WBRT than after SRS alone (7.8% vs 32.4%, respectively; difference, − 24.6%; 95% CI, − 35.7 to − 13.5%; *p* < 0.001). Lester et al. [9] showed that patients presenting with symptomatic brain recurrences would have worse clinical outcomes and create more costs for a healthcare system in comparison to asymptomatic patients with recurrences detected with routine surveillance imaging. Therefore, the importance of frequent surveillance imaging emerges with those concerns [10]. The National Comprehensive Cancer Network (NCCN) guidelines for limited numbers of BMs recommend follow-up (FU) with MRI every 2–3 months in the first year and thereafter every 4 to 6 months indefinitely [11]. However, there is little data about the actual impact of regular FU with MRI.

The aim of this retrospective, single center study is to report the potential benefit of regular MRI FU as well as the oncological outcomes and toxicity rates in patients with one to three BMs, who received surgery of at least one BM and post-operative local radiation treatment (SRS or hypo-fractionated stereotactic radiotherapy (HFSRT)) to the resection cavity and SRS to the remaining BMs.

## Patients and methods

### Patients

Medical records of patients with resected BMs and up to two unresected metastases were reviewed. Patients were treated between 2010 and 2015 at the Bern University Hospital with surgery of at least one BM followed by post-operative local radiation treatment (SRS or HFSRT) to the resection cavity and SRS to the remaining BMs. Eligibility criteria included patients older than 18 years with any non-CNS primary tumor histology and stage, Eastern Cooperative Oncology Group (ECOG) performance status 0–2, and the resected tissue had to be confirmed as metastatic histologically, consistent with a non-CNS primary site. Patients were excluded if they had prior cranial radiotherapy (RT), fractionation was > 10 and if there was no foreseen MRI FU or no available FU data. Patient data included: age, gender, performance status (ECOG score), graded prognostic assessment (GPA) score, primary tumor histology, number of BMs and location within the brain, if the BMs were symptomatic at diagnosis (yes vs. no) and if progressive or new neurological symptoms appeared after RT (yes vs. no), pre-RT resection status (gross total resection vs. subtotal resection), extracranial tumor disease status (continuously evaluated during the course of the disease), initial brain metastasis velocity (iBMV, according to Soike et al. [12]), brain metastasis velocity (BMV, according to Ferris et al. [13]), use of any systemic treatment, use and modality (surgery, radiosurgery and WBRT) of salvage treatment, site and timing of salvage therapy, date of every MRI FU and if MRI FU at 3 months and/or ≥ 1 MRI per 180 days was performed (yes vs. no).

### Stereotactic radiotherapy

According to institutional guidelines, the resection cavities were treated using robotic (Cyberknife, Accuray, Sunnyvale, USA) and Linac (Novalis, BrainLAB, Munich, Germany) based SRS, if the volume of the resection cavity was < 15 cm^3^ and HFSRT if the volume of the resection cavity was > 15 cm^3^. It was at the physician’s discretion to deviate from this standard procedure and to adapt the fractionation scheme according to the clinical situation in exceptional cases. The synchronous unresected metastases were treated with SRS alone. Patients were immobilized in supine position on the treatment table, using a commercial stereotactic mask fixation system in conjunction with the iPlan (BrainLAB, Munich, Germany) and Multiplan (Accuray, Sunnyvale, USA) treatment planning system. Target volumes and organs-at-risk (OAR) were delineated using postcontrast thin-slice (1 mm) gadolinium-enhanced T1-weighted and T2-weighted axial MRI sequences fused with thin-slice (0.75 mm) planning computed tomography (CT) scans. Target delineation and dose prescriptions were based on international consensus guidelines [8, 14–16]. To better compare the different treatment regimens, we calculated the biologically effective dose (BED) for each cavity depending on the number of fractions (Fx) and the single dose (SD) with a tumor α/β ratio of 10 [17]:

$$ \mathrm{BED}=\mathrm{Fx}\times \mathrm{SD}\times \left(1+\mathrm{SD}/10\right) $$

The following RT parameters were recorded: date of RT, device used for RT, fractionation, single dose, total dose, minimum target dose, maximum target dose, mean target dose, prescription isodose, planning target volume (PTV), gross tumor volume (GTV), margin and conformality index (CI). Radiotherapy outcome measures included local control (LC), distant brain control (DBC) and OS.

### Follow-up and toxicity assessment

After SRS/HFSRT, all patients were followed at 3-month intervals with neurological assessment. Serial brain MRI performance was individually decided by the treating physician. This was the standard of care in our hospital during that period of time. All observed adverse events were graded according to the National Cancer Institute’s Common Terminology Criteria for Adverse Events, version 4.0 (CTCAE-NCI v.4.0).

### MRI follow-up

Performing the first MRI scan 3 months after RT and/or performing ≥1 MRI per 180 days was defined as regular MRI FU. If MRI FU was performed, we have defined local failure (LF) as the development of new nodular contrast enhancement in the surgical bed compared with the baseline postoperative MRI. Local failure for unresected metastases was defined as an increase in size of more than 25%. Distant brain failure (DBF) was defined as the development of new, non-contiguous lesions in either MRI- or CT-follow-up. Leptomeningeal disease was diagnosed by imaging results consistent with this condition (either local or diffuse leptomeningeal disease) and rated as DBF. Diagnoses of tumor progression or radiation necrosis (RN) were determined based on histologic findings for patients who underwent surgical resection or by imaging using magnetic resonance spectroscopy (MRS). Lesions that were progressive on imaging and/or caused new neurological symptoms, but which dissolved without any further anti-cancer treatment, were considered RN rather than local failure. Neurological death was defined as uncontrolled intracranial tumor progress as well as new or progressive neurological symptoms prior to death. MRI data measures included: performance of a MRI FU 3 months post-RT (yes vs. no) and number of MRIs per 180 days post-RT until first salvage treatment. MRI outcome measures included the correlation between regular MRI FU and OS, time to any brain failure (ABF, i.e. LF or DBF), time to salvage treatment, symptom-free survival (SFS), deferment of WBRT and WBRT-free survival (WFS).

### Statistical analysis

Time to event data was calculated from the day of SRS or first day of HFSRT to the date of death or censored at last follow-up using the Kaplan-Meier method. Median follow-up time was determined with the reverse Kaplan-Meier method. Concerning OS and DBF, calculations were on a per patient basis (i.e. patients with more than one brain lesion were only considered once in the analysis) and for LF on a per cavity basis. For patients who had BMs treated sequentially, OS was considered from the date of the first SRS/HFSRT treatment. Univariate and multivariate Cox regression were used to investigate factors prognostic for LF, DBF and OS. For univariate analysis gender, age, primary tumor histology (breast, non-small cell lung, melanoma, colorectal and renal cell), residual tumor, GPA score, PTV >15cm^3^, BED < 40 Gy, MRI FU at 3 months and ≥ 1 MRI per 180 days were evaluated. MRI FU at 3 months and all covariates with a *p*-value of ≤0.1 in the univariate analysis concerning OS were intended for inclusion in the multivariate model. Group differences were assessed with the log-rank test. The association between regular MRI FU and time to WBRT was calculated with the Mann-Whitney-U-Test. A *p*-value ≤0.05 was considered statistically significant. All statistical analysis was completed using SPSS version 21 (IBM, USA).

### Ethics

All patients gave informed consent prior to initiation of treatment. Research ethics board approval was obtained for this analysis (Project ID: 2017–00033). This work is in accordance with the Declaration of Helsinki in its most recent version.

## Results

### Patient characteristics

Seventy-five patients were meeting the inclusion criteria of this study, comprising of 43 (57%) women and 32 (43%) men. Baseline characteristics are shown in Tables 1 and 2. A total of 77 resection cavities and 88 BMs were treated. Most patients (*n* = 63, 84%) had a single brain lesion and only 10 (13%) patients had synchronous BMs. Two (3%) patients received surgery and subsequent SRS/HFSRT to the surgical cavity for 2 BMs. Median follow-up time was 32.9 months (95% CI: 28.9–37 months). Median age at the time of RT was 62.9 years (range 33.3–79.2 years), while 54 (72%) patients had an ECOG score 0 and 58 (78%) patients had a GPA score ≥ 2.5. The most frequent primary tumor histologies were non-small cell lung cancer (*n* = 37, 49%) and melanoma (*n* = 13, 17%). At the time of diagnosis, 70 (93%) patients were symptomatic and total tumor resection was achieved in 60 (78%) metastases. A salvage treatment was administered to 31 (41%) patients. As first salvage treatment, surgery, SRS and WBRT were used for 10 (13%), 10 (13) and 11 (15%) patients, respectively. Median time from initial RT to first salvage treatment or last follow-up/death was 15.7 months (range, 2.5–30.2 months), while median time from first salvage treatment to death or last follow-up was 11.2 months (range, 0.3–33.7 months). There was a significant difference between groups concerning gender (*p* = 0.025), iBMV (*p* = 0.015) and BMV (*p* = 0.012) comparing patients that received ≥1 MRI vs. < 1 MRI per 180 days. Furthermore, patients that underwent MRI FU at 3 months had a significantly higher median iBMV value (*p* = 0.015).

**Table 1:** Patient characteristics

Characteristics	All patients	RT to cavity only	RT to cavity and in situ BM	*p*-value
Gender				0.855
Male	32 (43)	26 (35)	6 (8)	
Female	43 (57)	39 (52)	4 (5)	
Age at diagnosis (years)	62.9 (33.3–79.2)	63.3 (33.3–79.2)	58.2 (42.2–76.1)	0.436
Histology				0.262
Lung	37 (49)	35 (47)	2 (3)	
Breast	6 (8)	5 (7)	1 (1)	
Melanoma	13 (17)	11 (15)	2 (3)	
RCC	2 (3)	2 (3)	0 (0)	
Colorectal	8 (11)	6 (8)	2 (3)	
Other	9 (12)	6 (8)	3 (4)	
Location of cavity				0.441
Supratentorial	61 (79)	54 (70)	7 (9)	
Infratentorial	16 (21)	14 (18)	2 (3)	
Number of BM				< 0.001
1	63 (84)	63 (84)	NA	
2	11 (15)	2 (3)	9 (12)	
3	1 (1)	0 (0)	1 (1)	
Fractionation				0.517
SRS	60 (78)	53 (69)	7 (9)	
HFSRT	17 (22)	14 (18)	3 (4)	
ECOG score				0.305
0	54 (72)	48 (64)	6 (8)	
1	14 (19)	12 (16)	2 (3)	
2 or NA	7 (9)	5 (6)	2 (3)	
GPA score				0.002
3.5–4	23 (31)	23 (31)	0 (0)	
2.5–3	35 (47)	32 (43)	3 (4)	
0–2	13 (17)	8 (11)	5 (7)	
NA	4 (5)	2 (3)	2 (3)	
BM symptomatic initially				0.490
No or unknown	5 (7)	4 (5)	1 (1)	
Yes	70 (93)	61 (81)	9 (12)	
Residual tumor				0.143
No	60 (78)	54 (70)	6 (8)	
Yes	17 (22)	13 (17)	4 (5)	
Systemic cancer treatment				0.363
No or unknown	37 (49)	32 (43)	5 (7)	
Yes	38 (51)	33 (44)	5 (7)	
Cytotoxic	25 (33)	22 (29)	3 (4)	
Immunotherapy/targeted	6 (8)	4 (5)	2 (3)	
Combined	7 (9)	7 (9)	0 (0)	
Extracranial tumor status				0.462
Stable	27 (36)	25 (33)	2 (3)	
Progression	44 (59)	37 (49)	7 (9)	
Unknown	4 (5)	3 (4)	1 (1)	
Initial BMV	0.55 (0.05–11.4)	0.59 (0.05–11.4)	0.48 (0.13–4.93)	0.705
BMV	0.13 (0–84.4)	0 (0–64.9)	3.97 (1.34–84.4)	0.067
MRI follow-up at 3 months				0.112
No	35 (47)	28 (37)	7 (9)	
Yes	40 (53)	37 (49)	3 (4)	
No. of MRI per 180 days	1 (0–5.2)	1.1 (0–5.2)	0.3 (0–3.8)	0.083

Values represent numbers (percent) or median (range) if not specified otherwise

*RT* radiotherapy, *BM* brain metastasis, *RCC* renal cell carcinoma, *SRS* stereotactic radiosurgery, *HFSRT* hypo-fractionated stereotactic radiotherapy, *ECOG* Eastern Cooperative Oncology Group, *GPA* Graded Prognostic Assessment, *NA* not applicable, *BMV* brain metastasis velocity

**Table 2:** Patient characteristics regarding MRI follow-up

Characteristics	MRI FU at 3 months	No MRI FU at 3 months	*p*-value	≥1 MRI per 180 days	< 1 MRI per 180 days	*p*-value
Gender			0.662			0.025
Male	22 (29)	21 (28)		17 (23)	26 (35)	
Female	18 (24)	14 (19)		21 (28)	11 (15)	
Age at diagnosis (years)	60.6 (33.3–77.2)	64.4 (42.2–79.2)	0.137	61.1 (33.3–79.2)	62.9 (42.2–76.1)	0.511
Histology			0.079			0.222
Lung	25 (33)	12 (16)		23 (31)	14 (19)	
Breast	2 (3)	4 (5)		3 (4)	3 (4)	
Melanoma	4 (5)	9 (12)		5 (7)	8 (11)	
RCC	1 (1)	1 (1)		0 (0)	2 (3)	
Colorectal	2 (3)	6 (8)		2 (3)	6 (8)	
Other	6 (8)	3 (4)		5 (7)	4 (5)	
Location of cavity			0.473			0.953
Supratentorial	32 (42)	29 (38)		31 (40)	30 (39)	
Infratentorial	10 (13)	6 (8)		8 (10)	8 (10)	
Number of BM			0.463			0.333
1	35 (47)	28 (37)		34 (45)	29 (39)	
2	5 (7)	6 (8)		4 (5)	7 (9)	
3	0 (0)	1 (1)		0 (0)	1 (1)	
Fractionation			0.688			0.83
SRS	32 (43)	28 (36)		30 (39)	30 (39)	
HFSRT	10 (13)	7 (9)		9 (12)	8 (10)	
ECOG score			0.513			0.652
0	30 (40)	24 (32)		26 (35)	28 (37)	
1	8 (11)	6 (8)		8 (11)	6 (8)	
2 or NA	2 (3)	5 (7)		4 (5)	3 (4)	
GPA score			0.139			0.291
3.5–4	12 (16)	11 (15)		10 (13)	13 (17)	
2.5–3	22 (29)	13 (17)		21 (28)	14 (19)	
0–2	4 (5)	9 (12)		5 (7)	8 (11)	
NA	2 (3)	2 (3)		2 (3)	2 (3)	
BM symptomatic initially			0.387			0.331
No or unknown	3 (4)	2 (3)		3 (4)	2 (3)	
Yes	40 (53)	30 (40)		38 (51)	32 (43)	
Residual tumor			0.071			0.376
No	36 (47)	24 (31)		32 (42)	28 (36)	
Yes	6 (8)	11 (14)		7 (9)	10 (13)	
Systemic cancer treatment			0.573			0.978
No or unknown	21 (28)	16 (21)		19 (25)	18 (24)	
Yes	19 (25)	19 (25)		19 (25)	19 (25)	
Cytotoxic	14 (19)	11 (15)		12 (16)	13 (17)	
Immunotherapy/targeted	3 (4)	3 (4)		3 (4)	3 (4)	
Combined	2 (3)	5 (7)		4 (5)	3 (4)	
Extracranial tumor status			0.743			0.119
Stable	16 (21)	11 (15)		17 (23)	10 (13)	
Progression	22 (29)	22 (29)		18 (24)	26 (35)	
Unknown	2 (3)	2 (3)		3 (4)	1 (1)	
Initial BMV	0.72 (0.07–11.4)	0.39 (0.05–4.1)	0.015	0.7 (0.12–10.7)	0.39 (0.05–11.4)	0.015
BMV	0.65 (0–84.4)	0 (0–34.8)	0.139	1.33 (0–84.4)	0 (0–34.8)	0.012

Values represent numbers (percent) or median (range) if not specified otherwise

*FU* follow-up, *BM* brain metastasis, *RCC* renal cell carcinoma, *SRS* stereotactic radiosurgery, *HFSRT* hypo-fractionated stereotactic radiotherapy, *ECOG* Eastern Cooperative Oncology Group, *GPA* Graded Prognostic Assessment, *NA* not applicable, *BMV* brain metastasis velocity

**Fig. 1:**
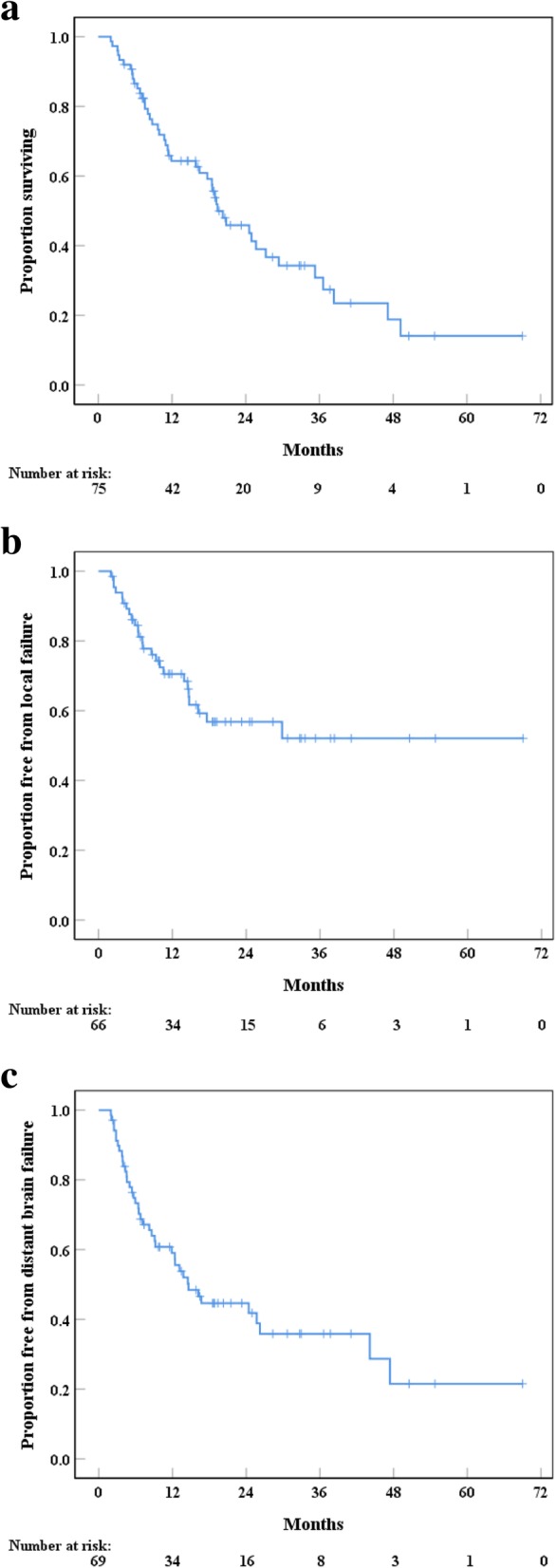
**a**–**c** Time from SRS or first day of HFRST to death or last follow-up (**a**), local failure (**b**) and distant brain failure (**c**)

**Fig. 2:**
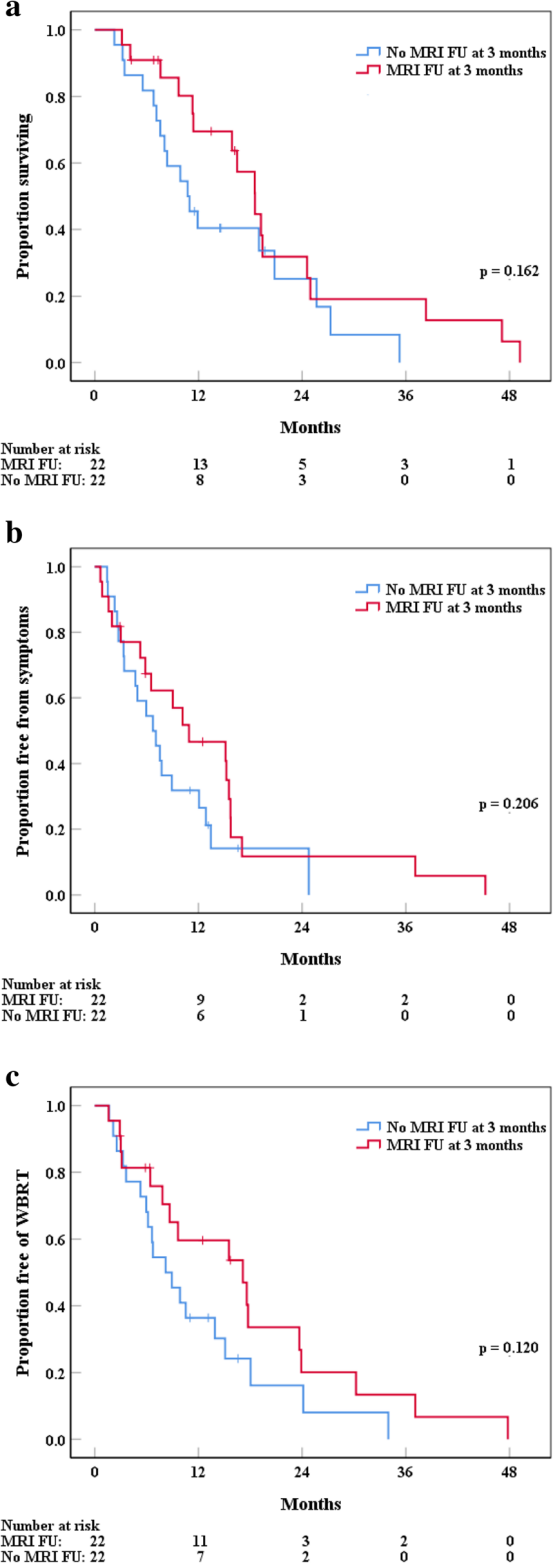
**a**-**c** Impact of MRI follow-up 3 months after radiotherapy (red graph) on overall survival (**a**), symptom-free survival (**b**) and WBRT-free survival (**c**) in a subgroup analysis of patients with extracranial progressive tumor disease

### Treatment characteristics

Dosimetric factors are summarized in Tables 3 and 4. The most commonly prescribed marginal dose to the resection cavity was 18 Gy (*n* = 36, 47%), most often prescribed to the 80% isodose (median prescription isodose: 80%, range 70–83%), resulting in a median mean dose of 18.3 Gy (range 16.6–24.5 Gy) for cavities treated with SRS. All HFSRTs were prescribed to the 80% isodose and 10 × 4 Gy was most frequently applied (*n* = 9, 12%), which resulted in a median mean dose of 39.9 Gy (range 24–45.7 Gy). Single-fraction SRS was administered to 60 (78%) cavities, while HFSRT was used for 17 (22%) cavities delivered in 5, 6 and 10 fractions to 4 (5%), 4 (5%) and 9 (12%) cavities, respectively. In the SRS-group, median resection cavity volume (GTV_res_) and median planning target volume (PTV_res_) were 4.6 cm^3^ (range 0.28–9.6 cm^3^) and 8.4 cm^3^ (range 1.8–21 cm^3^), respectively. In the HFSRT-group, median GTV_res_ was 15.3 cm^3^ (range 2.4–31.3 cm^3^) and median PTV_res_ 22.6 cm^3^ (range 4.9–45.5 cm^3^). A margin from GTV_res_ to PTV_res_ was applied to 74 (96%) cavities, while the median margin was 1.0 mm (range 0–2.5 mm).

**Table 3:** Treatment characteristics

Characteristics	SRS	HFSRT	All patients
Days from diagnosis to RT	34 (15–99)	35 (23–61)	34 (15–99)
Device used for RT			
Novalis (BrainLAB)	40 (52)	12 (16)	52 (69)
Cyberknife (Accuray)	20 (26)	5 (6)	23 (31)
GTV_res_	4.6 (0.3–9.6)	15.3 (2.4–31.3)	5.37 (0.3–31.3)
PTV_res_	8.4 (1.8–21)	22.6 (4.9–45.5)	10.6 (1.8–45.5)
PTV_res_ ≥ 15	6 (8)	14 (18)	20 (26)
PTV_res_ < 15	54 (70)	3 (4)	57 (74)
Cavity min. Dose	17.2 (6.3–19.7)	36.9 (19.3–40)	17.5 (6.3–40)
Cavity mean dose	18.3 (16.6–24.5)	39.9 (24–45.7)	19.2 (16.6–45.7)
Cavity max. Dose	19.1 (17–20.4)	40.4 (24.2–50)	20.4 (17–50)
Isodose line in %	80 (70–83)	80	80 (70–83)
Margin used for cavity			
0	3 (4)	0 (0)	3 (4)
0.5–1.5	40 (52)	12 (16)	52 (68)
1.8–2.5	17 (22)	5 (6)	22 (28)
Median margin for cavity	1 (0–2.5)	1 (1–2)	1 (0–2.5)
Conformality index	1.14 (1–1.68)	1.12 (1–1.67)	1.13 (1–1.68)
Patients with in situ BMs	7 (9)	3 (4)	10 (13)
GTV_in situ_	0.46 (0.19–1.67)	0.85 (0.06–2.18)	0.47 (0.06–2.18)
PTV_in situ_	0.48 (0.42–1.67)	1.05 (0.38–3.48)	0.51 (0.38–3.48)
Min. dose_in situ_	19.8 (18.6–21.5)	19.8 (19.6–38.3)	19.9 (18.6–38.3)
Mean dose_in situ_	22.7 (22–24.1)	21.4 (21.1–43.6)	22.6 (21.1–43.6)
Max. dose_in situ_	25 (22.5–25.3)	22.2 (21.8–47.5)	25 (21.8–47.5)

Values represent numbers (percent) or median (range) if not specified otherwise. Values for volume, margin and dose are reported in cm^3^, mm and Gy, respectively

*SRS* stereotactic radiosurgery, *HFSRT* hypo-fractionated stereotactic radiotherapy, *RT* radiotherapy, *GTV* gross tumor volume, *PTV* planning target volume, *Res* resection cavity, *BMs* brain metastases

**Table 4:** Single and multi-fractions schemes used for the cavities

Fx	SD [Gy]	TD [Gy]	BED [Gy]	Cavities
1	15	15	37.5	4 (5%)
	17	17	45.9	12 (16%)
	18	18	50.4	36 (47%)
	20	20	60	8 (10%)
5	5	25	37.5	1 (1%)
	6	30	48	3 (4%)
6	4	24	33.6	2 (3%)
	5	30	45	2 (3%)
10	4	40	56	9 (12%)

*Fx* fractions, *SD* single dose, *TD* total dose, *BED* biologically effective dose

### Local control and distant brain control

Overall, 77 resection cavities were evaluable on follow-up MR-imaging. Nine (12%) cavities could not be assessed for LC, due to death prior to the 3-months post-RT MRI FU scan (*n* = 3, 4%), availability of CT-scans only (*n* = 2, 3%) or complete loss to imaging follow-up (*n* = 4, 5%). While LF was seen in 25 (32%) resection cavities, DBF occurred in 36 (48%) patients. At one year, LC and DBC rates were 72% (95% CI: 60–83%) and 60% (95% CI: 49–72%), respectively (Fig. 1). Median time to DBF was 16.2 months (95% CI: 4.5–27.9 months), while no median time for LF was reached. No significant difference in LC and DBC was found for the different primary tumor histologies, margin and location of the BMs within the brain.

### Overall survival

At last FU, 30 (40%) patients were still alive. Median OS was 19.4 months (95% CI: 13.2–25.6 months) with 1- and 2-year OS of 64% (95% CI: 53–75%) and 44% (95% CI: 31–56%), respectively (Fig. 1). While median OS without any additional in situ BMs was 24.6 months (95% CI: 17.2–31.9 months), median OS for patients with 1 synchronous BM was 7.1 months (95% CI: 6.2–8.1 months) and the one patient with 2 synchronous BMs died 2 months after RT (*p* < 0.001). Patients with an ECOG score 0 had increased survival (24.6 months, 95% CI: 14.8–34.3 months) compared to patients with an ECOG score 1 (8.8 months, 95% CI: 0–23 months) and 2 (10.7 months, 95% CI: 0–23.3 months), however, no significance could be reached (*p* = 0.131). GPA score significantly correlated with OS: median survival for GPA 0–2 was 7.6 months (95% CI: 4.2–10.9 months), 20.8 months (95% CI: 13.8–27.8 months) for GPA 2.5–3 and 36.6 months (95% CI: 15.1–58.1 months) for GPA 3.5–4 (*p* = 0.001). Sixteen patients (21%) died of a neurological death. Histology of the primary tumor and receiving any systemic treatment had no impact on survival (*p* = 0.379). Similarly, no beneficial survival difference could be shown for immunotherapy and/or targeted therapy compared to cytotoxic chemotherapy (*p* = 0.186).

### Toxicity and radiation necrosis

Post-therapeutic fatigue was the most common adverse event and occurred in 10 (13%) patients. Nine (12%) patients reported post therapeutic headache and one patient (1%) suffered from vertigo. Hospitalization was necessary for 2 (3%) patients due to pneumonia and impossibility of mobilization, respectively. Radiation necrosis was verified histologically after surgery in 4 (5%) and suspected radiologically in 13 (17%) cavities.

### Factors prognostic for overall survival and intracranial failure

In multivariate analysis a PTV_res_ > 15 cm^3^ (HR 2.29, 95% CI: 1.22–4.31, *p* = 0.01), lower GPA score (0–2.0 vs. 2.5–3.0 vs. 3.5–4.0: HR 0.42, 95% CI: 0.26–0.69, *p* = 0.001) and residual tumor after surgery (HR 2.4, 95% CI: 1.29–5.64, *p* = 0.008) were associated with worse OS. A PTV_res_ > 15 cm^3^ was prognostic for increased LF in univariate (HR 3.42, 95% CI: 1.54–7.62, *p* = 0.003) and multivariate analysis (HR 3.2, 95% CI: 1.37–7.48, *p* = 0.007). Also, significantly higher LF rates for cavities which were irradiated with a BED of < 40 Gy were found in both uni- and multivariate analysis (HR 5.71, 95% CI: 2.05–15.9, *p* = 0.001). The two factors prognostic for DBF in multivariate analysis were PTV_res_ > 15 cm^3^ (HR 3.18, 95% CI: 1.34–6.16, *p* = 0.001) and lower GPA score (0–2.0 vs. 2.5–3.0 vs. 3.5–4.0: HR 0.46, 95% CI: 0.28–0.74, *p* = 0.001). The results of the univariate and multivariate analysis and a summary of outcomes are shown in Table 5 and 6.

**Table 5:** Uni- and multivariate analysis regarding local failure, distant brain failure and overall survival

	Univariate		Multivariate	
	HR (95% CI)	*p*-value	HR (95% CI)	*p*-value
Local failure				
Gender	1.26 (0.57–2.77)	0.573		
Age	1 (0.97–1.04)	0.912		
Histology	1.08 (0.83–1.4)	0.576		
Residual tumor	1.22 (0.42–3.58)	0.712	1.55 (0.47–5.1)	0.475
GPA score	0.66 (0.36–1.21)	0.18	0.82 (0.42–1.59)	0.552
PTV_res_ > 15 cm^3^	3.42 (1.54–7.62)	0.003	3.2 (1.37–7.48)	0.007
BED < 40 Gy	6.33 (2.5–15.9)	< 0.001	5.71 (2.05–15.9)	0.001
MRI FU at 3 months	0.97 (0.43–2.16)	0.936	0.96 (0.39–2.37)	0.926
≥ 1 MRI per 180 days	1.4 (0.63–3.14)	0.412		
Distant brain failure				
Gender	1.18 (0.62–2.24)	0.61		
Age	1.02 (0.99–1.05)	0.201		
Histology	1.07 (0.87–1.32)	0.535		
Residual tumor	1.26 (0.58–2.73)	0.559	1.52 (0.67–3.45)	0.322
GPA score	0.42 (0.26–0.67)	< 0.001	0.46 (0.28–0.74)	0.001
PTV_res_ > 15 cm^3^	3.7 (1.97–6.97)	< 0.001	3.18 (1.34–6.16)	0.001
BED < 40 Gy	2.1 (0.78–5.68)	0.143	1.31 (0.45–3.8)	0.623
MRI FU at 3 months	0.97 (0.52–1.81)	0.912	1.19 (0.59–2.38)	0.628
≥ 1 MRI per 180 days	1.44 (0.76–2.71)	0.262		
Overall survival				
Gender	1.57 (0.86–2.88)	0.141		
Age	1.01 (0.98–1.04)	0.637		
Histology	1.65 (0.96–1.41)	0.115		
Residual tumor	2.91 (1.58–5.38)	0.001	2.4 (1.29–5.64)	0.008
GPA score	0.39 (0.24–0.62)	< 0.001	0.42 (0.26–0.69)	0.001
PTV_res_ > 15 cm^3^	2.67 (1.47–4.85)	0.001	2.29 (1.22–4.31)	0.01
BED < 40 Gy	3.38 (1.31–8.7)	0.012	2.28 (0.82–6.33)	0.113
MRI FU at 3 months	0.8 (0.44–1.45)	0.461	1 (0.52–1.92)	0.993
≥ 1 MRI per 180 days	1.5 (0.83–2.72)	0.183		

*HR* hazard ratio, *CI* confidence interval, *GPA* Graded Prognostic Assessment, *PTV*_*res*_ planning target volume, *BED* biologically effective dose, *MRI FU* magnetic resonance imaging follow-up

Analyzing group differences with the log-rank test revealed significant shorter time to DBF (8.2 months, 95% CI: 0–22.1 months, vs. 24.4 months, 95% CI: 11.5–37.3 months, *p* = 0.009) for patients with synchronous in situ BMs, as well as patients with progressive extracranial tumor disease (11.1 months, 95% CI: 6.4–15.7 months vs. no median reached for patients with stable extracranial tumor disease, *p* = 0.001). Additionally, patients with progressive extracranial tumor status had decreased survival with a median OS of 16.5 months (95% CI: 7.1–25.8 months, *p* = 0.001), while no median OS value was reached for patients with stable extracranial tumor status. Patients with a BMV greater than the median had significantly reduced OS compared to the group with a BMV lower than the median (18.5 months, 95% CI: 9.9–27.1 months vs. 47.1 months, 95% CI: 21.2–73.1 months, *p* < 0.001) as well as reduced SFS (7.5 months, 95% CI: 5.1–10 months vs. 15.5 months, 95% CI: 8.5–22.5 months, *p* < 0.001) and reduced WFS (13.4 months, 95% CI: 4.5–22.2 months vs. 34.5 months, 95% CI: 30.8–52.5 months, *p* < 0.001). However, OS (*p* = 0.486), SFS (*p* = 0.834) and WFS (*p* = 0.319) was not decreased for patients with an iBMV greater than the median. Furthermore, patients which suffered from new or progressive neurological symptoms had significantly decreased survival (*p* = 0.012): One-year and 2-year survival rates were 57% (95% CI: 42–71%) and 37% (95% CI: 22–52%) for symptomatic patients and 86% (95% CI: 72–100%) and 69% (95 CI: 47–90%) for asymptomatic patients.

### Impact of regular MRI follow-up

Thirty-eight (51%) patients received ≥1 MRI per 180 days and 40 (53%) patients underwent MRI FU at 3 months. Concerning OS, no significant correlation between MRI FU at 3 months (*p* = 0.462) or ≥ 1 MRI per 180 days (*p* = 0.954) could be seen. Median SFS and median WFS were 10.2 months (95% CI: 5.7–14.7 months) and 17.7 months (95% CI: 13.2–22.3 months), respectively. Overall, MRI FU at 3 months had no significant impact on SFS (*p* = 0.613), WFS (*p* = 0.407), deferment of WBRT (*p* = 0.955), time to salvage treatment (*p* = 0.441), time to ABF (*p* = 0.492) and survival after salvage treatment (*p* = 0.885). Similarly, there was no significant correlation between ≥1 MRI per 180 days and SFS (*p* = 0.196), WFS (*p* = 0.308), deferment of WBRT (*p* = 0.268), time to salvage treatment (*p* = 0.054), time to ABF (*p* = 0.175) and survival after salvage treatment (*p* = 0.549). Increased OS was seen in a subgroup analysis of patients with progressive extracranial tumor disease, which had MRI FU at 3 months (18.5 months, 95% CI: 14.7–22.4 months vs. 10.7 months, 95% CI: 6.8–14.7 months, *p* = 0.162), although this result did not translate into statistical significance. Additionally, extracranially progressive patients with MRI FU at 3 months tended to have prolonged SFS (10.9 months, 95% CI: 2.7–19.1 months vs. 6.7 months, 95% CI: 3.7–9.7 months, *p* = 0.206) and WFS (17.1 months, 95% CI: 7.6–26.7 months vs. 8.2 months, 95% CI: 4.6–11.8 months, *p* = 0.120), compared to patients without MRI FU at 3 months (Fig. 2). No impact on OS (*p* = 0.531), SFS (*p* = 0.479) or WFS (*p* = 0.156) was seen in another subgroup analysis of patients with an iBMV greater than the median that underwent MRI FU at 3 months. Similarly, performing an MRI FU at 3 months for patients with a BMV greater than the median had no effect on OS (*p* = 0.415), SFS (*p* = 0.166) or WFS (*p* = 0.354). On the other hand, patients with an iBMV greater than the median that received ≥1 MRI per 180 days seemed to have worse SFS (12 months, 95% CI: 5.8–18.2 months vs. 29.1 months, 95% CI: 8.2–45.2 months, *p* = 0.03), while there was no association with OS (*p* = 0.173) or WFS (*p* = 0.156). Also, for patients with a BMV greater than the median which underwent ≥1 MRI per 180 days significantly reduced OS (11.9 months, 95% CI: 5.5–18.3 months vs. 25.7 months. 95% CI: 13.4–38 months, *p* = 0.032) and WFS (7.8 months, 95% CI: 3.4–12.3 months vs. 24.1 months, 95% CI: 8.7–39.5 months, *p* = 0.03), but no association with SFS (*p* = 0.126) was seen. In uni- and multivariate analysis regarding OS, LF and DBF, no significant results were seen for MRI FU at 3 months or ≥ 1 MRI per 180 days (Table 6).

**Table 6:** Summary of outcomes

Outcome	Definition	Result
Overall survival	Survival after SRS/HFSRT	19.4 months (95% CI: 13.2–25.6 months)
Local control	Absence of new nodular contrast enhancement in the surgical bed compared with the baseline postoperative MRI.	1 year rate: 72% (95% CI: 60–83%)
Distant brain control	Absence of new, non-contiguous lesions in either MRI- or CT-follow-up. Leptomeningeal disease was rated as DBF.	1 year rate: 60% (95% CI: 49–72%),
Radiation necrosis	Determined based on histologic findings (after surgery) or magnetic resonance spectroscopy (MRS).	17 (22%)
Neurological death	Uncontrolled intracranial tumor progress or new/progressive neurological symptoms prior to death	16 (21%)
Time to any brain failure	Time after initial SRS/HFSRT to local failure or distant brain failure	13.9 months (95% CI: 10.7–17.1 months)
Symptom-free survival	Survival after initial SRS/HFSRT without new or progressive neurological symptoms	10.2 months (95% CI: 5.7–14.7 months)
WBRT-free survival	Survival after initial SRS/HFSRT without undergoing WBRT	17.7 months (95% CI: 13.2–22.3 months)
Deferment of WBRT	Postponing of WBRT (through regular MRI FU)	MRI FU at 3 months: *p* = 0.955 ≥1 MRI per 180 days: *p* = 0.268.
Time to salvage treatment	Time after initial SRS/HFSRT to first salvage treatment	15.7 months (range, 2.5–30.2 months)
Survival after salvage therapy	Survival after first salvage therapy	11.2 months (range, 0.3–33.7 months)

*SRS* stereotactic radiosurgery, *HFSRT* hypo-fractionated stereotactic radiotherapy, *MRI* magnetic resonance imaging, *FU* follow-up, *CT* computed tomography, *DBF* distant brain failure, *MRS* magnetic resonance spectroscopy, *WBRT* whole brain radiotherapy

## Discussion

Although adjuvant WBRT leads to increased DBC and less neurological deaths [7, 18, 19], no survival benefit could be shown compared to adjuvant SRS to the surgical cavity. Brown et al. [8] recently showed in a randomized phase III trial improved cognitive outcomes with postoperative SRS in comparison with WBRT, without OS differences. However, numerous authors pointed out the increased risk of DBF when WBRT is omitted [7, 8, 14, 20–23] and emphasized the need for close brain MRI FU in such patient cohorts [14, 24]. With this current single-center retrospective study, we report the impact of regular MRI FU for patients with 1 to 3 BMs treated with surgery followed by SRS or HFSRT to the surgical cavity. To our knowledge, this is the first report focussing on the impact of regular MRI FU in patients with a limited number of brain metastases treated with surgery and focal RT. Our general study results about oncological outcome are well comparable with the existing literature. We report excellent median OS of 19.4 months (95% CI: 13.2–25.6 months) in our cohort, compared with 10.9 to 17 months described in literature [8, 14, 20, 24]. Actuarial LC and DBC one year after RT were 72% (95% CI: 60–83%) and 60% (95% CI: 49–72%), respectively. These rates coincide with the literature, where reported 1-year LC is 71 to 90% and 1-year DBC 36 to 53% [14, 20–22, 24]. We were also able to confirm in multivariate analysis, as described in the literature [20–22, 24–27], that the following factors were prognostic for significantly worse survival: a PTV > 15 cm^3^ (*p* = 0.01), residual tumor after resection (*p* = 0.008) and a lower GPA score (0–2.0 vs. 2.5–3.0 vs. 3.5–4.0, *p* = 0.001). Likewise, additional in situ BMs (*p* < 0.001) and progressive neurological symptoms (*p* = 0.012) were associated with worse survival. Against our hypothesis, regular MRI FU in such a cohort, consisting mainly of patients with a single and resected BM, surprisingly did not result in increased OS, SFS, WFS or deferment of WBRT. However, patients with extracranial progressive tumor disease which received MRI FU 3 months after SRS/HFSRT had increased OS (*p* = 0.162), SFS (*p* = 0.203) and WFS (*p* = 0.120), compared to patients without MRI FU at 3 months, although these differences between groups did not translate into statistical significance. Nevertheless, one could hypothesize, that patients with extracranially progressive disease may profit more from regular MRI FU, because of the increased metastatic spread efficiency with an uncontrolled primary tumor status [28]. Likewise, having more than one BM at diagnosis could be an indication of a further progressed primary tumor. Several studies stated that apart from systemic progression, a primary pathology of melanoma and ≥ 3 BMs are prognostic for DBF [14, 21, 29]. While tumor histology (*p* = 0.535) had no impact on DBF in our cohort in univariate analysis, additional unresected BMs (*p* = 0.009) and uncontrolled primary tumor status (*p* = 0.001) were significantly associated with shorter time to DBF. To develop the aforementioned hypothesis further, patients with more than one BM and/or melanoma tumor histology might therefore benefit more from regular MRI FU. In contrast, one might speculate that concerning OS, SFS and WFS, patients with a single and resected BM and/or controlled primary tumor status do not profit as much from regular MRI FU, which could be the reason why we were not able to show a significant impact of regular MRI FU in our cohort (84% of our patients had a single and resected BM).

Farris et al. [13] introduced the novel metric BMV, which is defined as the cumulative number of new BM that developed over time since first SRS in years. The authors were able to show that a lower BMV correlated significantly with increased OS, increased freedom from WBRT and a reduced incidence of neurological death. This is of special interest, as BMV might help to decide if a patient should receive either salvage SRS or WBRT in case of second DBF. In addition, Soike et al. [12] were able to show that the metric iBMV (which is defined as number of BMs at initial SRS devided by time (years) from initial cancer diagnosis to first SRS) correlated with BMV and OS. Thus, iBMV could serve as a metric to help triage patients to initial SRS or WBRT. In our cohort we can confirm that a BMV greater than the median is associated with worse OS (*p* < 0.001), as well as worse SFS (*p* < 0.001) and WFS (*p* < 0.001). On the other hand, iBMV had no impact on OS, SFS or WFS. Median BMV value was 0.13 in our cohort and hence noticeably lower compared to a median value of 5.5 in the cohort of Farris et al. [13]. In the study of Soike et al. [12], the iBMV value differed significantly depending on the primary tumor. The iBMV tends to be higher for tumor histologies that spread faster or more likely to the brain. On that account, we believe that iBMV is a surrogate marker for tumor biology and therefore also for histology. Since tumor histology had no impact on OS in our cohort, it is well explicable that iBMV was also not associated with OS. Concerning OS, SFS and WFS no benefit was seen for patients with an iBMV or BMV greater than the median, which underwent regular MRI FU. In fact, patients with an iBMV greater than the median that received ≥1 MRI per 180 days had significantly worse SFS (*p* = 0.03) and patients with a BMV greater than the median which received ≥1 MRI per 180 days had significantly reduced OS (*p* = 0.032) and WFS (*p* = 0.03). There seems to be a discrepancy concerning the benefit of regular MRI FU for patients with an increased risk of potentially developing new BMs (i.e. extracranial progressive tumor disease) and for patients that effectively develop more new BMs (i.e. increased BMV). It has to be noted that the median iBMV was a significantly higher in the patient cohort that underwent MRI FU at 3 months and those that received ≥1 MRI per 180 days (see Table 2, for both *p* = 0.015). Similarly, a significant higher median BMV value was seen for patients that underwent ≥1 MRI per 180 days (*p* = 0.012). A reason for this group differences might be selection bias, as the performance of regular MRI FU was at the physician’s discretion. Therefore, in our retrospective study, it is probable that patients with primary tumor histologies that are known to predominantly metastasize to the brain (i.e. lung cancer) are more likely to be selected into the “regular MRI FU group”. Another reason for the imbalance is probably due to the fact that regular MRI FU shortens the time to detect new BMs, which results in a higher BMV. All above mentioned limitations indicate that the results concerning iBMV/BMV and regular MRI FU have limited validity in our cohort. Also, the counterintuitive and illogical results concerning regular MRI FU and increased iBMV/BMV indicate that the impact of regular MRI FU might be of less significance than previously presumed in a cohort consisting of patients with a good performance status and with a single and resected BM. Therefore, a prospective evaluation of the impact of regular MRI FU is needed.

Nevertheless, to be able to deliver local radical therapies effectively (initially or as salvage treatment), precise tumor imaging with brain MRI is paramount [14, 22, 23, 28]. In a retrospective review of patients receiving salvage SRS after prior brain RT, Kurtz et al. [30] were able to show that especially younger patients with controlled extracranial tumor disease and durable response to initial brain RT presumably profit the most from salvage SRS. The authors concluded that this circumstance implies the need for intracranial monitoring for these patient groups. Additionally, preventing new or progressing symptoms and delivering SRS initially (and thus deferring WBRT and its neurotoxic effects with an increased risk of DBF) are other mentioned reasons in literature proposing the need of regular MRI FU [7]. Chang et al. [23] stated in their randomized trial that the benefit of close imaging surveillance is supported by the fact that 18 out of 21 patients with DBF were asymptomatic when recurrences were discovered in MRI FU. Furthermore, it is crucial to bear in mind that neurocognitive decrease can result from both WBRT and recurrent brain metastases. In order to reduce the impact of recurrent metastases on neurocognition, many authors advocate performing regular MRI FU, as salvage surgery and SRS can be repeated [14, 23, 31]. Preventing symptoms caused by recurring BMs is not only desirable from a clinical point of view, but possibly also for the health care system. On average, management of symptomatic patients noticeably drains more on the resources, as they are more likely to undergo neurosurgical interventions and tend to have longer hospital stays, whereas asymptomatic patients can be managed with outpatient SRS more frequently [9]. In a retrospective study of patients with mostly oligometastatic brain disease (89% of the patients had ≤3 BMs) who received upfront SRS, Lester et al. [9] compared the clinical and economical outcomes of asymptomatic and symptomatic patients. The authors created a model of 5000 patients who underwent regular MRI FU and evaluated its possible financial benefit based on the survival rate of asymptomatic patients, time to DBF, MRI costs, estimated detection rate of new lesions and estimated costs of managing symptomatic and asymptomatic metastases. Based on this model, they estimated that surveillance brain imaging after radiosurgery could save insurers an average of $1326 per patient.

Nevertheless, since there are no prospective trials that analyzed the impact of regular MRI FU, the actual benefit remains unclear. In addition, Yiu et al. [32] stated that close surveillance imaging might also induce anxiety and lower the patient’s quality of life. In their feasibility survey about the benefit of MRI FU, the authors were able to show that within 6 months 10 (45%) out of 22 patients agreed to participate in the experimental arm without regular MRI surveillance. A prospective cohort study would therefore be feasible.

We could not confirm a significant correlation between OS and ECOG/KPS score in our cohort. A reason for increased OS in our cohort might be due to stricter patient selection: Sixty-eight (91%) patients had an ECOG of 0–1, 63 (84%) had a single BM and 58 (78%) a GPA score ≥ 2.5. Referring to the recent phase III trial of Brown et al. [8], the reported median OS was 12.2 months, while 90% had an ECOG 0–1 and 23% of the patients treated with SRS after surgery had > 1 BMs. Similarly, Choi et al. [14] reported a median OS of 17 months, while only 63% had a single BM and 24% had a GPA score > 3.

Therefore, some important limitations should be acknowledged in our study. Being of retrospective nature, this study is limited by inherent biases. Any conclusions revealed here are hypothesis-generating and, as such, need to be validated within a prospective study. There may be a selection bias in our patient cohort, as only patients with adjuvant SRS were taken into account. Patients with poorer prognosis and hence poorer performance score were less likely to receive SRS. A reporting bias is also probable, as patients in poor general health condition were less likely to undergo complete follow-up scheme or were lost to follow-up and therefore excluded from the study due to lack of data. Regular MRI FU was often not performed strictly according to the guidelines, which resulted in a large heterogeneity of different FU intervals. Also, since the performance of regular MRI FU was at the physician’s discretion, a bias in patient selection in this regard seems probable. Therefore, a prospective study assessing the impact of regular MRI FU is needed.

## Conclusion

Our results regarding oncological outcomes consist with the current data from the literature. Overall, regular MRI FU did not result in increased OS, SFS, WFS or deferment of WBRT in our cohort consisting mainly of patients with a single and resected BM. Based on a subgroup analysis concerning OS, SFS and WFS, we found that patients with progressive extracranial tumor disease might profit more from regular MRI FU. The impact of regular MRI FU needs prospective evaluation.

## Abbreviations

ABF: Any brain failure
BED: Biologically effective dose
BMs: Brain metastases
BMV: Brain metastasis velocity
CI: Conformality index
CNS: Central nervous system
CT: Computed tomography
CTCAE-NCI: National Cancer Institute’s Common Terminology Criteria for Adverse Events
DBC: Distant brain control
DBF: Distant brain failure
ECOG: Eastern Cooperative Oncology Group
FU: Follow-up
Fx: Fractions
GPA: Graded prognostic assessment
GTV: Gross tumor volume
HFSRT: Hypo-fractionated stereotactic radiotherapy
iBMV: Initial brain metastasis velocity
LC: Local control
LF: Local failure
MRI FU: Magnetic resonance imaging follow-up
MRS: Magnetic resonance spectroscopy
NCCN: National Comprehensive Cancer Network
OAR: Organs-at-risk
OS: Overall survival
PTV: Planning target volume
RCC: Renal cell carcinoma
RN: Radiation necrosis
RT: Radiotherapy
SD: Single dose
SFS: Symptom-free survival
SRS: Stereotactic radiosurgery
WBRT: Whole brain radiotherapy
WFS: WBRT-free survival
